# Inferring Functional Neural Connectivity with Phase Synchronization Analysis: A Review of Methodology

**DOI:** 10.1155/2012/239210

**Published:** 2012-04-22

**Authors:** Junfeng Sun, Zhijun Li, Shanbao Tong

**Affiliations:** ^1^Med-X Research Institute, School of Biomedical Engineering, Shanghai Jiao Tong University, Shanghai 200030, China; ^2^Department of Automation, School of Electronic Information and Electrical Engineering, Shanghai Jiao Tong University, Shanghai 200240, China

## Abstract

Functional neural connectivity is drawing increasing attention in neuroscience research. To infer functional connectivity from observed neural signals, various methods have been proposed. Among them, phase synchronization analysis is an important and effective one which examines the relationship of instantaneous phase between neural signals but neglecting the influence of their amplitudes. In this paper, we review the advances in methodologies of phase synchronization analysis. In particular, we discuss the definitions of instantaneous phase, the indexes of phase synchronization and their significance test, the issues that may affect the detection of phase synchronization and the extensions of phase synchronization analysis. In practice, phase synchronization analysis may be affected by observational noise, insufficient samples of the signals, volume conduction, and reference in recording neural signals. We make comments and suggestions on these issues so as to better apply phase synchronization analysis to inferring functional connectivity from neural signals.

## 1. Introduction

Segregation and integration are two fundamental organization principles of neural systems in brain [1]. The neural organization can be investigated with functional neural connectivity in both local and global brain regions. The connectivity in local regions reflects specialized functions of local cortex, while the connectivity among spatially separated brain regions plays important roles in advanced cognitive function [2–7]. Various measures, such as phase synchronization index (PSI), mutual information, correlation coefficient, coherence, and partial directed coherence, have been applied to inferring neural connectivity among brain units at multiple temporal and spatial scales with neural signals including electroencephalography (EEG), magnetoencephalography (MEG), functional magnetic resonance imaging, and local field potential [8–13]. These measures can be classified into several families such as correlation/coherence family, phase synchronization family, and Granger causality family. The measures in the same family usually yield results with strong correlation to each other and thus could provide little additional information on neural connectivity, while some measures belonging to different families may have weak correlation to each other in inferring neural connectivity, which implies that they each could characterize interdependence of signals in different aspects [13].

Among these measures, PSI quantifies the relationship between instantaneous phases (IP, represents the rhythm of oscillation or signal wave) of coupled systems/brain units but neglects the effect of their amplitude. PSI has been demonstrated to be effective in inferring neural connectivity, especially when the connectivity is too weak to be detected by other measures [8, 14]. Note that there are other types of synchronization, such as complete synchronization and generalized synchronization [14, 15]. But most of them are defined for theoretical models of coupled oscillators and difficult to be applied to neural signal analysis. The coupled systems/units are claimed to be in phase synchronization (PS) when the difference of IPs is bounded with respect to time. PS is ubiquitous in both natural and man-made systems, such as neural oscillations [4, 16], coupled chaotic oscillators [17], chaotic laser arrays [18], and electrochemical oscillations [19]. In addition, two metrics of PS, that is, the phase-lock interval and the lability of global synchronization, hold power law probability distributions for both simulation systems (i.e., the Ising model and the Kuramoto model) and brain networks at a broadband frequency. These results imply that the IP association among multiple units or brain regions hold the property of criticality [20].

PSI, also called phase-locking value in literature, is proposed to quantify the level of PS and has been applied to a wide range of neuroscience research [3, 5]. For example, PSI has been used to examine the alternation of cortical connectivity prior to seizures [21] and neuronal synchrony after ischemic stroke [22], to identify mental states in brain-computer interface [23, 24], to investigate cognitive dysfunctions of mental disorder [25], and to gain new strategies for clinical treatment [22, 26]. However, to get a reliable estimation of PSI from observed neural signals is not so easy, especially when the signals are with a small number of samples and/or contaminated by noise [10, 11, 27, 28]. Note that, in literature, other measures, such as phase clustering index, used different definition of phase, which is not within the framework of IP [29, 30]. In this paper, we review the methodology of PS analysis in inferring functional neural connectivity from the following aspects.

(1) IP definition. To detect PS in a pair of signals, various IP definitions have been proposed. Most of these IP definitions are based on particular transforms, such as the Hilbert transform [17] and the wavelet transform [31]. But actually, these IP definitions can be unified into one framework which defines IP with specific filter applied to signals [32].
(2) PSI and its significance test. We will introduce two commonly used PSIs, which are based on entropy [2, 33] and circular statistics [34, 35], respectively. We will further introduce several strategies to provide significance test for the estimated PSI.
(3) Practical problems in PS detection. The estimation of PSI is affected by observational noise, volume conduction, the number of samples in observed signals, reference, and other factors. We will summarize the advances on these issues and give suggestions for a better PS detection.
(4) Extensions of PS analysis. Lots of extensions/variations of PS analysis have been proposed for multitrial signals and multivariate signals. We will give a brief overview of these methods and some comments to their applications in neural signal analysis.

## 2. Definition of Instantaneous Phase

**Figure 1:**
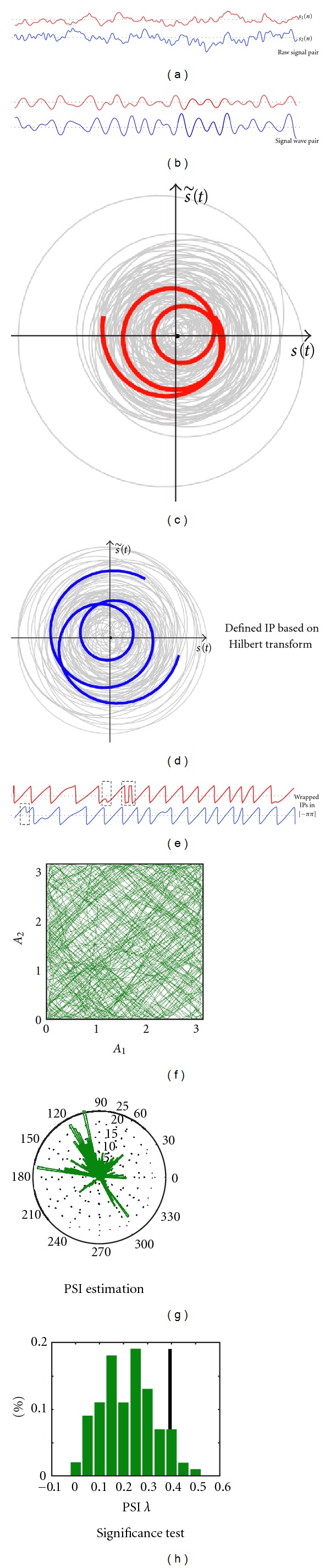
Schematic diagram of phase synchronization (PS) analysis. For broadband raw signals (a), a bandpass filter is first applied to extracting signal waves in specific frequency band (b). Then analytic signals of signal waves may be defined based on the Hilbert transform ((c) and (d)), and the argument of the analytic signals are defined as instantaneous phase (IP) of the corresponding signal waves. The IPs could be wrapped into the range [−*π*, *π*] (e). In some cases as marked by dotted rectangle in (e), the estimated analytic signal [$s(t)+j\tilde{s}(t)$] may be ill-defined due to noisy data and does not always rotate counterclockwise around origin in the complex plane, resulting in non-monotonic IP “jump” at the time when the trajectory of analytic signal crosses through the origin. Signals with too many IP “jumps” are not suitable for PS analysis. With the differences of IPs which are wrapped in the range [−*π*, *π*], PS index (PSI), which quantifies the level of PS, could be estimated according to the distribution of IP difference (g). In addition, significance test could provide a significance threshold (the black bar in (h)) for estimated PSI. If the estimated PSI is greater than the threshold, then the corresponding signal wave pair is claimed to be in significant PS with a certain confidence level. For some cases, the amplitudes of analytic signals may be rather weakly correlated (f), but the corresponding PSI is with relatively large value. For the case in (f) and (g), the correlation coefficient between the amplitudes (i.e., *A*_1_ versus *A*_2_) of two signal waves is −0.07, while the corresponding MPC-based PSI is 0.44.

Before estimating the level of PS, the IP of each signal should be defined (Figure 1). The most basic definition of IP is based on the Hilbert transform [14, 17, 36], which can be directly applied to coherent signals (i.e., narrow band signals with one prominent spectral component). But, for noncoherent data such as raw neural signals, this IP definition may yield negative instantaneous frequency (IF, defined as the derivative of IP with respect to time), which is physically meaningless [37–39]. One way is to process noncoherent signal with a specific narrow bandpass filter. An alternative way is to define IP based on other transforms such as the wavelet transform [18, 19, 31, 40]. PS detection based on these IP definitions has been compared numerically with both simulation data and experimental signals, yielding similar connectivity [10, 11]. In addition, an analytical study on these IP definitions has unified them into one framework which defines IP with a specific bandpass filter applied [32]. In this section, we will briefly introduce issues on IP definition based on results in [32].

### 2.1. Definitions of Instantaneous Phase

#### 2.1.1. IP Definition Based on the Hilbert Transform

For a given signal *s*(*t*), its analytic signal is defined as

(1) $$s^{\left(h\right)}\left(t\right)=s\left(t\right)+j\tilde{s}\left(t\right),$$

where

(2) $$\tilde{s}\left(t\right)=ℋ\left[s\left(t\right)\right]=\frac{1}{\pi }\text{P}.\text{V}.\overset{\infty }{\underset{-\infty }{\int }}\frac{s\left(\tau \right)}{t-\tau }d\tau$$

is the Hilbert transform of *s*(*t*) (here P.V. means that the integral is taken in the sense of the Cauchy principal value). Then the IP of *s*(*t*) is defined as

(3) $$\phi^{\left(h\right)}\left(t\right)=\arg\left[s^{\left(h\right)}\left(t\right)\right]=\arctan\frac{\tilde{s}\left(t\right)}{s\left(t\right)}.$$

In the frequency domain, *s* ^(*h*)^(*t*) appears as *S* ^(*h*)^(*f*) = *S*(*f*)*B* ^(*h*)^(*f*), where *S*(*f*) is the Fourier transform of *s*(*t*) and

(4) $$B^{\left(h\right)}\left(f\right)=\{\begin{array}{cc} 2, & \text{if}\;f>0\\ 1, & \text{if}\;f=0\\ 0, & \text{if}\;f<0 \end{array}$$

is the Fourier transform of *b* ^(*h*)^(*t*) = *δ*(*t*) + *j*(1/*πt*). Then in numerical implementation, the estimation of analytic signal *s* ^(*h*)^(*t*) can be obtained by the inverse Fourier transform of *S* ^(*h*)^(*f*). In the time domain, *s* ^(*h*)^(*t*) can be expressed as the convolution of *s*(*t*) with the complex-response filter *b* ^(*h*)^(*t*); that is,

(5) $$s^{\left(h\right)}\left(t\right)=s\left(t\right)*b^{\left(h\right)}\left(t\right).$$

#### 2.1.2. IP Definition Based on Gaussian Filter

Another method defines IP as

(6) $$\phi^{\left(g\right)}\left(t\right)=\arg\left[s^{\left(g\right)}\left(t\right)\right],$$

where

(7) $$s^{\left(g\right)}\left(t\right)=s\left(t\right)*b^{\left(g\right)}\left(t\right)$$

is the convolution of *s*(*t*) with a narrow-band Gaussian filter $b^{\left(g\right)}(t)=\left(1/\sqrt{2\pi }T\right)e^{-t^{2}/(2T^{2})}e^{j2\pi f_{n}t}$, which is shifted by the nominal frequency *f*_*n*_ [18]. In the frequency domain, *s* ^(*g*)^(*t*) can be expressed as *S* ^(*g*)^(*f*) = *S*(*f*)*B* ^(*g*)^(*f*), where *B* ^(*g*)^(*f*) = *e* ^−2*π*^2^*T*^2^(*f*−*f*_*n*_)^2^^. PS analysis based on this IP definition has successfully detected PS in coupled laser arrays, which was not revealed by IP defined with the Hilbert transform [18]. The possible reason is that the Gaussian filter extracts the components of the laser data in a particular frequency band which are in PS, while PS analysis based on the Hilbert transform does not use filter to extract the components, and thus the underlying PS level is submerged by noise and components in other bands. 

#### 2.1.3. IP Definition Based on the Wavelet Transform

With the Gabor wavelet *ψ*(*t*) = *g*(*t*)*e* ^*j*2*πνt*^, IP is defined as

(8) $$\phi^{\left(w\right)}\left(t\right)=\arg\left[s^{\left(w\right)}\left(t\right)\right],$$

where

(9) $$s^{\left(w\right)}\left(t,a\right)=s\left(t\right)*b^{\left(w\right)}\left(t\right)$$

is the convolution of *s*(*t*) with *b* ^(*w*)^(*t*) = *f*_*n*_^1/2^ *g*(*f*_*n*_ *t*)*e* ^*j*2*πf*_*n*_*t*^, and *g*(*t*) = (*T* ^2^ *π*)^−1/4^ *e* ^−*t*^2^/(2*T*^2^)^ is the envelope [31]. The difference between IP definitions based on the Gaussian filter and on the wavelet transform lies in that the filters *b* ^(*w*)^(*t*) and *b* ^(*g*)^(*t*) are with Gaussian windows of different amplitudes and width; that is, *b* ^(*w*)^(*t*) is scaled by *f*_*n*_. In the frequency domain, *g*(*t*) appears as *G*(*f*) = (4*πT* ^2^)^1/4^ *e* ^−2*π*^2^*f*^2^*T*^2^^, and *b* ^(*w*)^(*t*) is *B* ^(*w*)^(*f*) = *f*_*n*_^−1/2^ *G*((*f*/*f*_*n*_) − 1).

#### 2.1.4. Unified Framework for IP Definitions

The IP definitions discussed above all can be expressed as the argument of the signal after a specific filter. In other words, these IP definitions can be unified into one common framework [32]; that is,

(10) $$\begin{array}{c} \phi^{\left(b\right)}\left(t\right)=\arg\left[s^{\left(b\right)}\left(t\right)\right], \end{array}$$

where

(11) $$\begin{array}{c} s^{\left(b\right)}\left(t\right)=s\left(t\right)*b\left(t\right). \end{array}$$

Here *b*(*t*) = *g*(*t*)*e* ^*j*2*πf*_*n*_*t*^ is a filter with envelope $g(t)=\left(1/\sqrt{2\pi }T\right)e^{-t^{2}/(2T^{2})}$, nominal frequency *f*_*n*_, and response duration *T*. The filter assures that the extracted signal wave is coherent. *s* ^(*b*)^(*t*) is analytic in an asymptotic sense as the bandwidth of *b*(*t*) is smaller than 2*f*_*n*_ [36, 41, 42]. This method is exactly the one based on a Gaussian filter when the envelope *g*(*t*) is a Gaussian function. Filter *b*(*t*) can be expressed as

(12) $$B\left(f\right)=\overset{\infty }{\underset{-\infty }{\int }}b\left(t\right)e^{-j2\pi ft}dt=G\left(f-f_{n}\right)$$

in the frequency domain, where *G*(*f*) = *e* ^−2*π*^2^*f*^2^*T*^2^^ is the Fourier transform of *g*(*t*).

The analytic signal *s* ^(*b*)^(*t*) = *s*(*t*)∗*b*(*t*) can be interpreted as a combination of the Hilbert transform and a real bandpass filter. Let *s* ^(*r*)^(*t*) = *s*(*t*)∗[*g*(*t*)cos⁡(2*πf*_*n*_ *t*)], where *g*(*t*)cos⁡(2*πf*_*n*_ *t*) is the real part of *b*(*t*). Then *s* ^(*r*)^(*t*) appears as

(13) $$S^{\left(r\right)}\left(f\right)=S\left(f\right)\left[\frac{1}{2}G\left(f+f_{n}\right)+\frac{1}{2}G\left(f-f_{n}\right)\right]$$

in the frequency domain. With (4), the analytic signal of *s* ^(*r*)^(*t*) can be obtained by performing the inverse Fourier transform to *S* ^(*r*)^(*f*)*B* ^(*h*)^(*f*); that is,

(14) $$\begin{array}{cc} & \;{ℱ}^{-1}\left[S^{\left(r\right)}\left(f\right)B^{\left(h\right)}\left(f\right)\right]\\ & \;\;={ℱ}^{-1}\left\{S\left(f\right)\left[\frac{1}{2}G\left(f+f_{n}\right)+\frac{1}{2}G\left(f-f_{n}\right)\right]B^{\left(h)(f\right)}\right\}\\ & \;\;={ℱ}^{-1}\left[S\left(f\right)G\left(f-f_{n}\right)\right]\\ & \;\;=s\left(t\right)*\left[g\left(t\right)e^{j2\pi f_{n}t}\right]\\ & \;\;=s^{\left(b\right)}\left(t\right), \end{array}$$

where *ℱ* ^−1^(·) denotes the inverse Fourier transform operator.

Beside IP definitions within this framework, there are IP definitions proposed in other aspects [43, 44]. For example, the IP and PSI based on Rihaczek distribution are more robust to noise than the method based on the wavelet transform [43].

### 2.2. Constraints for IP Definition

For a real signal *s*(*t*) = *ℛ*[*A*(*t*)*e* ^*jϕ*(*t*)^], its imaginary counterpart *ℐ*[*A*(*t*)*e* ^*jϕ*(*t*)^] is usually unobservable, where *ℛ*(·) and *ℐ*(·) denote the real and the imaginary parts of the complex variable (·), respectively. Generally, the imaginary part is estimated from *s*(*t*) by a certain operation; that is, $ℐ[A(t)e^{j\phi (t)}]=\tilde{ℋ}[s(t)]$. Among various operators $\tilde{ℋ}(\cdot )$ proposed, the Hilbert transform *ℋ*(·) is the only one that satisfies the following three conditions [45].

(i) The associated amplitude *A*(*t*) is continuous and differentiable.
(ii) The IPs of signals *s*(*t*) and *c* · *s*(*t*) is the same; that is, the operator should possess the property $\tilde{ℋ}[c\cdot s(t)]=c\cdot \tilde{ℋ}[s(t)]$, where *c* is a constant.
(iii) For any constant amplitude *A* > 0, frequency *ω* > 0, and phase *ψ*, the operator satisfies $\tilde{ℋ}[A\cos(\omega t+\psi )]=A\sin(\omega t+\psi )$.

In addition, the Bedrosian theorem gives more constraints on IP definition based on the Hilbert transform [36, 42]. This theorem states that the relation

(15) ℋ[l(t)h(t)]=l(t)ℋ[h(t)]

holds for a low-frequency term *l*(*t*) and a high-frequency term *h*(*t*) whose spectra do not overlap in the frequency domain. For real signal *s*(*t*) = *A*(*t*)cos⁡*ϕ*(*t*), *s* ^(*q*)^(*t*) = *A*(*t*)*e* ^*jϕ*(*t*)^ is its *quadrature model*. It seems that *ϕ*(*t*) in this model is a good candidate for definition of IP. However, it is difficult to estimate *A*(*t*) and *ϕ*(*t*) from only the observable signal *s*(*t*) with this model. There is a difference between *s* ^(*q*)^(*t*) and the analytic signal *s* ^(*h*)^(*t*) (1). This difference approaches zero in an asymptotic sense as *A*(*t*) and cos⁡*ϕ*(*t*) fulfill the Bedrosian theorem [42]. Note that the Bedrosian theorem sets a constraint to the signal, which is similar to the second condition; that is, $\tilde{ℋ}[c\cdot s(t)]=c\cdot \tilde{ℋ}[s(t)]$, that set on the operator. For noncoherent signal, bandpass filter *b*(*t*) is usually applied, so that the extracted signal wave *s* ^(*b*)^ = *s*(*t*)∗*b*(*t*) is coherent and fulfills the Bedrosian theorem. The effective bandwidth of filter *b*(*t*) is $\Delta f=1/\left(2\sqrt{2}\pi T\right)$, which should be less than 2*f*_*n*_ [32]. As the filter *b*(*t*) becomes narrower close to delta function (i.e., *δ*(*f* − *f*_*n*_)) in the frequency domain, the IF (1/2*π*)(*dϕ* ^(*b*)^(*t*)/*dt*) of the components in the pass band approaches the nominal frequency *f*_*n*_ in an asymptotic sense [46].

Generally, to infer functional connectivity with PS analysis, we would recommend to define IP by combining the Hilbert transform with specific bandpass filter; that is, a bandpass filter is first applied to extracting the neural signal waves in specific frequency band, and then IP is defined based on the Hilbert transform.

## 3. Phase Synchronization Analysis

### 3.1. Phase Synchronization Indexes

Let *ϕ*_1_(*t*) and *ϕ*_2_(*t*) denote the cumulative IP of signals observed from two coupled units, respectively. Then the signal pair is said to be in *p* : *q* PS when the inequality |*pϕ*_1_(*t*) − *qϕ*_2_(*t*)| < const. holds, where *p* and *q* are positive integers. In this paper, we focus on the case of 1 : 1 PS, which is defined based on samples of one trial in the time domain. Most conclusions for 1 : 1 PS can be easily extended to the case of *p* : *q* PS [47, 48]. In neuroscience research, the estimated PSI is usually taken as one kind of functional connectivity in neural signals. Here we introduce two PSIs which have been commonly used to quantify the functional connectivity of neural signals. They are based on entropy [2, 33] and circular statistics [34, 35], respectively. Let *s*(*n*) denote *s*(*n*Δ*t*), that is, the observation of *s*(*t*) at time *n*Δ*t*, and let $\hat{\phi }(n)$ denote the estimation of *ϕ*(*t*) at time *n*Δ*t*, where Δ*t* is the sampling interval.

(i) The entropy-based PSI is estimated by
  (16) $$\rho =\frac{\left(Q_{\max}-Q\right)}{Q_{\max}},$$
  where *Q* = −∑_*i*=1_^*K*^ *P*_*i*_ln⁡(*P*_*i*_) is the entropy of distribution $P(\hat{\varphi })$, $\hat{\varphi }(n)={\hat{\phi }}_{1}(n)-{\hat{\phi }}_{2}(n)$, *Q*_max⁡_ = ln⁡*K*, and *K* is the number of bins of distribution [2, 33].
(ii) The mean phase coherence (MPC) of IP difference *φ*(*t*) = *ϕ*_1_(*t*) − *ϕ*_2_(*t*) is another commonly used PSI. It is defined as *λ* = |*E*[*e* ^*jφ*^]| and can be estimated via
  (17) $$\begin{array}{cc} \lambda & =\frac{1}{N}\left|\overset{N-1}{\underset{n=0}{\sum }}e^{j\hat{\varphi }(n)}\right|, \end{array}$$
  where ${\left\{\hat{\varphi }(n)\right\}}_{n=0}^{N-1}$ is the estimation of *φ*(*t*) and *N* is the number of samples.

These two PSIs quantify how concentrated the distribution of IP difference is. Their values are in [0, 1], with PSI = 1 implying that the signal pair is with exact rhythm locking and PSI = 0 indicating no PS at all. Beside these two indexes, other measures, such as the index based on conditional probability [2] and the index based on mutual information [49], have also been applied to the quantification of relationship between IPs. More discussions on PSI can be found in [8].

### 3.2. Significance Tests for PSI

In inferring neural connectivity, a highly concerned question is whether the estimated PSI could indicate that the signal pair is with significant connective strength or not. This question is also concerned in brain network research, as brain network is constructed by setting edges between nodes (i.e., brain regions) with connective strength over a predefined threshold. Accumulated studies show that both functional and structural brain networks is of small-worldness with sparse edge number [28, 50–55]. The topology of brain networks are dependent on the number of edges, that is, the number of connectivity with significantly big strength. One way to decide the value of this common threshold is based on significance test for estimated PSI [8, 34, 49, 56]. Here we introduce three different strategies in providing significance threshold for PS analysis.

#### 3.2.1. Artificial Surrogate Tests

The first strategy is based on artificial surrogate data. Surrogate methods usually produce artificial data by randomizing the concerned property but keeping as much as possible other properties of the original signal [49, 57]. Then whether the original signal possesses the concerned property could be tests with the artificial surrogate data. Various surrogate methods have been proposed [8, 57–61]. For example, surrogate method based on autoregressive model generates surrogate realizations by fitting an autoregressive model of the original signals and using independent white noise as the inputs to the model. The surrogate data generated by this method are linear stochastic processes and have the same power spectra of the original signals [57, 60]. In another study, a method named twin surrogate is proposed based on the recurrence properties. It is demonstrated to be suitable to provide significance test for PS in Rössler oscillators [61].

In [28], four different surrogate methods, which generate artificial surrogate data by shuffling the rank order, the phase spectra, the IP of original EEG signals, are compared in significance test for PS analysis. Results show that the phase-shuffled surrogate method is workable for PS analysis. This method generates surrogate data by shuffling the phase spectra of original signal but keeping the amplitude spectra unchanged. Let {*S*(*k*)}_*k*=0_^*N*−1^ denote the discrete Fourier transform of original signal {*s*(*n*)}_*n*=0_^*N*−1^; that is,

(18) $$S\left(k\right)=\overset{N-1}{\underset{n=0}{\sum }}s\left(n\right)e^{-j2\pi kn/N},\;k=0,1,\ldots ,N-1.$$

Then a surrogate realization of {*s*(*n*)} is generated by the inverse discrete Fourier transform of $\tilde{S}(k)$; that is,

(19) $$\tilde{s}\left(n\right)=\frac{1}{N}\overset{N-1}{\underset{k=0}{\sum }}\tilde{S}\left(k\right)e^{j2\pi kn/N},\;n=0,1,\ldots ,N-1,$$

where

(20) $$\tilde{S}\left(k\right)=\left|S\left(k\right)\right|e^{j\nu (k)},$$

with a uniform random sequence {*ν*(*k*)}_*k*=0_^*N*−1^ [57, 58].

**Figure 2:**
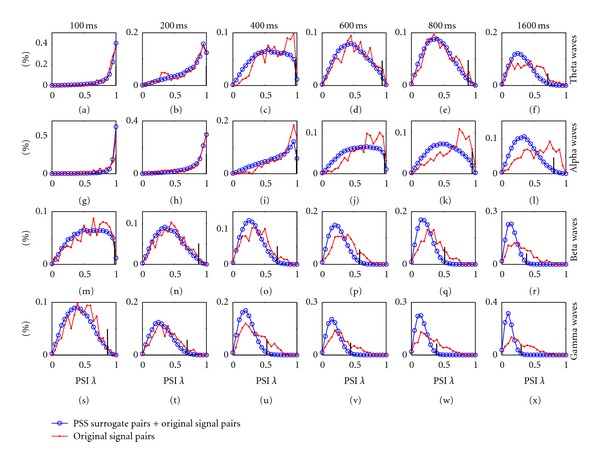
Histogram of the phase synchronization indexes (PSI) *λ* for 435 original EEG signal pairs and their 435 × 99 = 43 065 phase-shuffled surrogate pairs for one subject. The results for the theta, alpha, beta, and gamma waves of six different duration, that is, 100 ms, 200 ms, 400 ms, 600 ms, 800 ms, and 1600 ms, are presented. For each subfigure, all the PSIs *λ* of original EEG signal pairs and their phase-shuffled surrogate pairs are sorted in ascending order, and the black bar indicates the value (*x*-axis) of the one at the rank of 95% of all the PSIs. In other words, the black bar indicates the threshold of 95% level of significance for the estimated PSIs of original EEG signal pairs in each case.

A (1 − *α*) × 100% level of significance corresponds to a probability *α* of a false rejection. A larger number of surrogate realizations could offer a greater power in discrimination [57]. In [28], 99 surrogate pairs are generated for each original EEG signal pair. Then if the PSI of one original signal pair is larger than the 5th biggest in all the 100 PSIs (i.e., the PSIs of the original signal pair and its 99 surrogate pairs), the original signal pair is claimed to be in PS with a 95% level of significance.  Figure 2 shows the histogram of the MPC-based PSIs *λ* for 435 original EEG signal pairs and their phase-shuffled surrogate pairs for one subject, where one common significance threshold is estimated for all 435 original signal pairs in each case.

#### 3.2.2. Analytical Significance Test

The second strategy is based on the distribution test of IP series [34, 56]. When the distribution of PSI is well approximated by an analytical model, a significance threshold then could be offered analytically. Empirical distributions of IP difference of coupled Rössler systems have been tested under the assumption that IP obeys specific distributions and the IPs of different samples are independent [56]. But this is applicable only for special cases, as the distribution of IP difference may vary with respect to different dynamical systems. In another study, the IP difference is formulated as an increment process under the assumption that the increments could be represented by an *α*-mixing process. A theoretical significance level is proposed for the MPC-based PSI. Simulation results show that the significance level is workable when the time series is with enough samples and two dependent parameters could be reliably estimated [34].

#### 3.2.3. Surrogate Test Based on Intersubject Signal Pairs

The third strategy is based on the assumption that the neural signals of different subjects are independent. In [28], intersubject EEG signal pair denotes two EEG signals from two different subjects, and intra-subject EEG signal pair denotes two EEG signals from the same subject. Then under this assumption, the intersubject EEG signal pairs can be used as surrogate pairs for intra-subject EEG signal pairs. Compared with above artificial surrogate data, intersubject surrogate pairs seem to possess more inherent features of EEG signals but with no association between each other. Then significance test based on intersubject EEG signal pairs may be used as a standard criterion in evaluating the performance of artificial surrogate methods. Significance test with this strategy shows that the histogram of the MPC-based PSIs *λ* for intersubject surrogate pairs and intra-subject EEG signal pairs is similar to that based on the phase-shuffled surrogate method (Figure 2) [28], and the significance threshold suggested by intersubject surrogate pairs is close to that by the phase-shuffled surrogate method. This implies that the phase-shuffled surrogate method is workable in providing significance test for PS analysis. In our opinion, more studies on significance test for PS analysis are needed, and the studies combined the three different strategies introduced above would be promising when they may reach consistent results. 

## 4. Problems in Phase Synchronization Detection

In real applications, the observed neural signals are more or less contaminated by noise, and the samples collected are usually limited. Obviously, the observational noise will degrade the estimation of PSI and even submerge non-trivial PS in neural signals. In addition, the MPC-based PSI is a biased estimator, which implies that the reliability of functional connectivity inferred by it will decrease as the samples collected in signals are insufficient. To get a reliable PS detection in real applications, we must take the issues into consideration. In this section, we would discuss challenges of PS analysis in practice. In particular, we would introduce the advances on the effect of noise in PS detection, the influence of signal duration and estimation bias of PSI, the effect of volume conduction, and the influence of reference in PS analysis.

### 4.1. Effect of Noise in PS Detection

The effect of noise in PS detection has been examined by both numerical computation [10, 11, 27, 33, 62, 63] and analytical study [32]. A bandpass prefiltering can suppress the effect of noise but may introduce spurious connectivity as well [33]. In this section, we mainly review a theoretical study on the effect of noise in IP estimation and PS detection based on the unified framework of IP definition [32].

Let *s*(*t*) = *x*(*t*) + *w*(*t*) denote the noisy signal, where *x*(*t*) is the clean signal and *w*(*t*) is the additive noise. The analytic signal of *s*(*t*) can be defined with a bandpass filter *b*(*t*); that is,

(21) $$\begin{array}{cc} s^{\left(b\right)}\left(t\right) & =s\left(t\right)*b\left(t\right)\\ & =x\left(t\right)*b\left(t\right)+w\left(t\right)*b\left(t\right)\\ & =A_{x}\left(t\right)e^{j\phi_{x}^{\left(b\right)}(t)}+w^{\left(b\right)}\left(t\right), \end{array}$$

where *w* ^(*b*)^(*t*) = *w*(*t*)∗*b*(*t*). Let $\theta (t)={\hat{\phi }}_{s}^{\left(b\right)}(t)-\phi_{x}^{\left(b\right)}(t)$ denote the estimate error of IP due to the noise term *w*(*t*), where ${\hat{\phi }}_{s}^{\left(b\right)}(t)$ denotes the estimate of *ϕ*_*x*_^(*b*)^(*t*) from the noisy signal *s*(*t*). It has been proved that the distribution of *θ*(*t*) is a normal distribution; that is, *θ* ~ *N*(0, *σ*_*θ*_^2^),

(22) $$p\left(\theta \right)={\left(\sqrt{2\pi }\sigma_{\theta }\right)}^{-1}e^{-\theta^{2}/\left(2\sigma_{\theta }^{2}\right)},$$

when the instantaneous signal-to-noise ratio, defined as *r* ^(*b*)^(*t*) = *A*_*x*_^2^(*t*)/[2*σ*_*w*^(*b*)^_^2^] in the pass band, is larger than 5. Here *σ*_*w*^(*b*)^_^2^ is the variance of *w* ^(*b*)^(*t*), and *σ*_*θ*_ = *σ*_*w*^(*b*)^_/*A*_*x*_(*t*). The wrapped *θ*, that is, Θ = *θ*(mod⁡2*π*), obeys the wrapped normal distribution, $\Theta \sim \tilde{N}(0,\sigma_{\theta }^{2})$ [35, 64],

(23) $$p\left(\Theta \right)=\frac{1}{\sqrt{2\pi }\sigma_{\theta }}\overset{\infty }{\underset{k=-\infty }{\sum }}e^{-{\left(\Theta +2k\pi \right)}^{2}/\left(2\sigma_{\theta }^{2}\right)}.$$

For the MPC-based PSI, *λ* = |*E*[*e* ^*jφ*^]|, the effect of noise appears as

(24) $$\hat{\lambda }=e^{-(\sigma_{\theta_{1}}^{2}+\sigma_{\theta_{2}}^{2})/2}\lambda ;$$

that is, the noise introduces a degrading factor, *e* ^−(*σ*_*θ*_1__^2^+*σ*_*θ*_2__^2^)/2^, into the true index *λ*. This factor is determined by only the level of in-band noise. If *σ*_*θ*_ = *σ*_*w*^(*b*)^_/*A*_*x*_ in (22) is not a constant, *θ* actually obeys a conditional distribution, and (22) turns out to be

(25) $$p\left(\theta \;|\;\sigma_{\theta }\right)={\left(\sqrt{2\pi }\sigma_{\theta }\right)}^{-1}e^{-\theta^{2}/(2\sigma_{\theta }^{2})},\;\sigma_{\theta }>0.$$

For observed signal {*s*(*n*)}, the distribution of IP error is a scale mixture of normal distributions (SMN) with different variances. If the probability density function of {*σ*_*θ*_(*n*)} is known, the empirical distribution of IP error {*θ*(*n*)} can be approximated as

(26) $$p_{m}\left(\theta \right)=\overset{K}{\underset{k=1}{\sum }}p\left(\theta \;|\;\sigma_{k}\right)\pi_{k},$$

where {*π*_*k*_}_*k*=1_^*K*^ are the respective empirical probabilities which are estimated from {*A*_*x*_(*n*)} [65]. Note that *A*_*x*_(*n*) is the instantaneous amplitude of the clean signal, and thus it is difficult to obtain its distribution with only the observed noisy signal {*s*(*n*)}. Simulations are performed by considering the SMN of the IP error as a normal distribution with constant standard deviation *σ*_*w*^(*b*)^_/max⁡{*A*_*x*_(*n*)}; that is, *σ*_*θ*_ = *σ*_*w*^(*b*)^_/max⁡{*A*_*x*_(*n*)}, and computational outputs support the theoretical results (24). When the in-band noise level is not so high (signal-to-noise ratio greater than 10 dB), the estimated PSI is not much affected by observational noise [32].

### 4.2. Influence of Signal Duration and Estimation Bias

Another question in PS analysis is how long a epoch of signals is enough to get a reliable quantification of neural connectivity? One microstate of EEG signals usually lasts 50 to 200 ms [3, 9, 66]. However, various durations (from 100 ms to 10 ms) of EEG signals have been used in examining functional connectivity [12, 24, 67, 68]. A comprehensive study has been performed regarding this issue based on both surrogate tests and intersubject EEG surrogate test [28]. Results show that a duration of EEG waves of 3 ~18 wave cycles is suitable for PS analysis. The value of PSI is not only determined by the relationship between two IP sequences but also highly affected by the duration of signals used for PSI estimation. Too short duration of signals will result in too large PSI, while too long duration of signals will yield too small PSI, both of which may not offer a good indication on the level of PS (Figure 2) [28].

In another study, the IP difference of a signal pair with no synchrony is assumed to obey uniform distribution. Under this assumption, the expected value of the MPC-based PSI is

(27) $$E\left\{\lambda \right\}\approx \frac{1}{\sqrt{N}},$$

where *N* is the number of samples used for PSI estimation [43, 69]. This means that the MPC-based PSI *λ* is a positively biased estimators for finite sample sizes [70]. More samples used will result in a less biased PSI. Then even for the same duration of signals, signals observed with higher sampling rate will yield a better estimation of the MPC-based PSI than those with lower sampling rate. The effect of sampling rate on both the MPC-based PSI and the entropy-based PSI has been evaluated. Results demonstrate that the MPC-based PSI *λ* is less affected by sampling rate than the entropy-based PSI and thus is recommended for PS analysis of EEG signals measured with low sampling rate [28].

In many experimental studies, the number of samples or trials of measured data is limited. Bias will be unavoidable in PSI estimation due to insufficient samples. In addition, even when the observed signals are with sufficient samples, they may be nonstationary. In this case, we still want to estimate PSI with samples in short time window, so as to obtain functional connectivity with a certain degree of temporal resolution. Regarding this problem, a new measure called pairwise phase consistency has been proposed [71]. This measure is defined as the mean of the cosine of IP difference across all given signal pairs; that is,

(28) $$\xi =\frac{2}{N\left(N-1\right)}\overset{N-1}{\underset{i=1}{\sum }}\overset{N}{\underset{k=i+1}{\sum }}\cos\left(\phi_{i}-\phi_{k}\right).$$

Pairwise phase consistency quantifies the similarity of relative IP among trials or samples and is bias-free. In addition, it is demonstrated that pairwise phase consistency is equivalent to the squared MPC-based PSI in population statistic [71].

### 4.3. Influence of Volume Conduction

The activities of a single source within the brain can be observed by many sensors on the scalp [72]. This is usually referred as volume conduction. Then the PSI between signals measured by different sensors, especially by spatially adjacent sensors, may be a trivial artefact due to volume conduction, but not a true interaction of the underlying brain activities [73]. There are two ways to tackle this problem. One way is to estimate the true sources of underlying brain activities with observed EEG/MEG signals using inverse method and then quantify the relationship of the estimated sources rather than the relationship of the observed signals. However, the true sources are usually not easy to be obtained by inverse methods.

An alternative way is based on the imaginary part of coherency of observed signals. We assume that two signals *s*_*i*_(*t*) and *s*_*j*_(*t*) collected by sensors *i* and *j* are from a linear superposition of *K* independent sources *x*_*k*_(*t*), and the mapping of each source to sensors is instantaneous with no distortion [74]. Then in the frequency domain, *s*_*i*_(*t*) can be expressed as

(29) $$S_{i}\left(f\right)=\overset{K}{\underset{k=1}{\sum }}a_{ik}X_{k}\left(f\right),$$

where *S*_*i*_(*f*) and *X*_*k*_(*f*) are the Fourier transform of *s*_*i*_(*t*) and *x*_*k*_(*t*), respectively, and *a*_*ik*_ are the contribution coefficient of *x*_*k*_(*t*) to *s*_*i*_(*t*). The cross-spectrum of *s*_*i*_(*t*) and *s*_*j*_(*t*) is

(30) $$\begin{array}{cc} C_{ij}\left(f\right) & =E\left\{S_{i}\left(f\right)S_{j}^{*}\left(f\right)\right\}\\ & =\overset{}{\underset{kk^{\prime }}{\sum }}a_{ik}a_{jk^{\prime }}E\left\{X_{k}\left(f\right)X_{k^{\prime }}^{*}\left(f\right)\right\}\\ & =\overset{}{\underset{kk^{\prime }}{\sum }}a_{ik}a_{jk^{\prime }}\delta_{kk^{\prime }}E\left\{{\left|X_{k}\left(f\right)\right|}^{2}\right\}\\ & =\overset{}{\underset{k}{\sum }}a_{ik}a_{jk}E\left\{{\left|X_{k}\left(f\right)\right|}^{2}\right\}, \end{array}$$

where *δ*_*kk*′_ denotes the Kronecker-delta function. Here *C*_*ij*_(*f*) is real, which implies that volume conduction of multiple sources strongly affects the real part of the cross-spectrum between *s*_*i*_(*t*) and *s*_*j*_(*t*) but does not affect the imaginary part [75, 76].

The complex coherency of *s*_*i*_(*t*) and *s*_*j*_(*t*) is defined as

(31) $$\Omega \left(f\right)=\frac{C_{ij}\left(f\right)}{{\left[C_{ii}\left(f\right)C_{jj}^{*}\left(f\right)\right]}^{1/2}}.$$

In [75], the imaginary part of *Ω*(*f*) is defined as a synchronization measure for *s*_*i*_(*t*) and *s*_*j*_(*t*) in the frequency domain. The mean of *Ω*(*f*) over all frequencies is equal to the mean of the cross-correlation of the corresponding analytic signals over time [76]; that is,

(32) $${\langle \Omega \left(f\right)\rangle }_{f}=\frac{{\langle A_{i}\left(t\right)A_{j}\left(t\right)e^{j(\phi_{i}(t)-\phi_{j}(t))}\rangle }_{t}}{{\left[{\langle A_{i}^{2}\left(t\right)\rangle }_{t}{\langle A_{j}^{2}\left(t\right)\rangle }_{t}\right]}^{1/2}},$$

where 〈·〉_*f*_ and 〈·〉_*t*_ denote the average, respect to frequency and time respectively. Then the imaginary part of 〈*Ω*(*f*)〉_*f*_ is

(33) $$ℐ\left[{\langle \Omega \left(f\right)\rangle }_{f}\right]=\frac{{\langle A_{i}\left(t\right)A_{j}\left(t\right)\sin\left[\phi_{i}\left(t\right)-\phi_{j}\left(t\right)\right]\rangle }_{t}}{{\left[{\langle A_{i}^{2}\left(t\right)\rangle }_{t}{\langle A_{j}^{2}\left(t\right)\rangle }_{t}\right]}^{1/2}}.$$

However, it has been demonstrated that the imaginary part of 〈*Ω*(*f*)〉_*f*_ is not a good index of PS as it depends on the amplitudes of signals [76, 77]. Further, a measure called phase lag index,

(34) $$\eta =\left|{\langle \mathrm{sign}\left[\phi_{i}\left(t\right)-\phi_{j}\left(t\right)\right]\rangle }_{t}\right|,$$

was proposed based on the consideration that “the existence of a consistent, nonzero phase lag between two times series cannot be explained by volume conduction from a single strong source” [76]. Recently, it is demonstrated that the performance of phase lag index could be degraded by small perturbations [70]. To deal with this problem, a weighted phase lag index is defined as

(35) $$\zeta =\frac{\left|E\left\{ℐ\left[S_{i}\left(f\right)S_{j}^{*}\left(f\right)\right]\right\}\right|}{E\left\{\left|ℐ\left[S_{i}\left(f\right)S_{j}^{*}\left(f\right)\right]\right|\right\}}.$$

Compared with phase lag index, the weighted phase lag index is demonstrated with “reduced sensitivity to additional, uncorrelated noise sources and increased statistical power to detect changes in phasesynchronization” [70].

### 4.4. Influence of Reference

The influence of reference on EEG signals is a long-lasting problem in quantifying functional connectivity [78–80]. Various reference strategies, such as bipolar EEG [81], average common reference EEG [82], and Laplacian EEG [73], have been proposed. However, the cautions on these reference strategies have been extensively reported as well [81, 83].

The influence of reference has been examined with both analytical analysis and computational simulation [68]. Let *u*(*t*) = *Vr*(*t*) denote the reference signal, where *V* > 0 is coefficient and *r*(*t*) is time-dependent. Let *y*_*i*_(*t*) denote the scalp signal measured by the *i*th sensor, and, *s*_*i*_(*t*) = *u*(*t*) − *y*_*i*_(*t*) denote the signal *y*_*i*_(*t*) rereferenced to *u*(*t*). For analytic signal *s*_*i*_(*t*) defined with the Hilbert transform, we have

(36) $$\begin{array}{cc} & \underset{V\to +\infty }{\lim}\phi_{s_{i}}\left(t\right)\\ & \;=\underset{V\to +\infty }{\lim}\arctan\frac{{\tilde{s}}_{i}\left(t\right)}{s_{i}\left(t\right)}\\ & \;=\underset{V\to +\infty }{\lim}\arctan\frac{\left(1/\pi \right)\text{P}.\text{V}.\int_{-\infty }^{+\infty }\left[\left(Vr\left(\tau \right)/t-\tau \right)-\left(y_{i}\left(\tau \right)/t-\tau \right)\right]d\tau }{Vr\left(t\right)-y_{i}\left(t\right)}\\ & \;=\underset{V\to +\infty }{\lim}\arctan\frac{\left(1/\pi \right)\text{P}.\text{V}.\int_{-\infty }^{+\infty }\left[\left(Vr\left(\tau \right)/t-\tau \right)-\left(y_{i}\left(\tau \right)/t-\tau \right)\right]d\tau }{Vr\left(t\right)}\\ & \;=\underset{V\to +\infty }{\lim}\arctan\frac{\left(1/\pi \right)\text{P}.\text{V}.\int_{-\infty }^{+\infty }\left(Vr\left(\tau \right)/t-\tau \right)d\tau }{Vr\left(t\right)}\\ & \;=\underset{V\to +\infty }{\lim}\arctan\frac{\left(1/\pi \right)\text{P}.\text{V}.\int_{-\infty }^{+\infty }\left(r\left(\tau \right)/t-\tau \right)d\tau }{r\left(t\right)}. \end{array}$$

Then we have

(37) $$\underset{V\to +\infty }{\lim}\lambda_{s_{1}s_{2}}=\underset{V\to +\infty }{\lim}\left|E\left\{e^{j[\phi_{s_{1}}(t)-\phi_{s_{2}}(t)]}\right\}\right|=1,$$

as lim⁡_*V*→+*∞*_ *ϕ*_*s*_1__(*t*) − lim⁡_*V*→+*∞*_ *ϕ*_*s*_2__(*t*) = 0. This result indicates that the coefficient *V* has a great influence on the MPC-based PSI *λ*. A sufficiently large *V* will lead to larger PSI *λ*_*s*_1_*s*_2__ for referential signals than that for nonreferential signals [68]. Simulation study demonstrates that the PSI *λ*_*s*_1_*s*_2__ of two referential signals may monotonically increase to 1 or decrease first and then increase to 1 as the coefficient *V* increases from 0 to *∞*. In addition, a method based on independent component analysis has been demonstrated to be an appropriate method to generate reference for EEG signals in quantifying connectivity [84].

## 5. Extensions of Phase Synchronization Analysis

Various extensions of PS analysis have been proposed to infer the relationship in multivariate or multitrial signals based on the concept of IP [85–90]. For example, a method named frequency flow analysis has been used to examine the global synchronization of multivariate signals [88]. If the IF, derived from IP, of each variable almost equals to the IFs of other variables in a certain frequency band for a duration, the multivariate signals are called in PS accordingly. In this section, we will further introduce two extensions/variations of PS analysis, that is, the trial-based PSI [85, 86] and the partial PSI [87].

A trial-based PSI is proposed to examine the variation of IP difference between signal channels across trials under repeated stimulus [85, 86]. For a data set of *K* trials and each trial with *N* samples, the trial-based PSI is defined as

(38) $$\lambda_{s_{i}s_{j}}\left(t\right)=\frac{1}{K}\overset{K}{\underset{k=1}{\sum }}e^{j[\phi_{s_{i}}(t,k)-\phi_{s_{j}}(t,k)]},$$

where *ϕ*_*s*_*i*__(*t*, *k*) and *ϕ*_*s*_*j*__(*t*, *k*) are the IPs of the channels *s*_*i*_ and *s*_*j*_ in the *k*th trial. The PSI *λ*_*s*_*i*_*s*_*j*__(*t*) measures the intertrial variability of IP difference between channels *s*_1_ and *s*_2_ at instant *t*. The PSI *λ*_*s*_*i*_*s*_*j*__(*t*) with value close to 1 implies that the IP difference varies little across trials at instant *t*, while the PSI *λ*_*s*_*i*_*s*_*j*__(*t*) with value close to zero means that the IP difference varies approximately uniformly across trials.

The pairwise PSI discussed above can infer the strength of connectivity in two signals but cannot indicate whether the connectivity is induced by the direct coupling between them or due to the indirect interaction mediated by other units. To deal with this problem, a measure called partial PSI is generalized from PS analysis following the idea of partial coherence [87, 91]. Combined with the pairwise PSI, the partial PSI can be used to distinguish the direct and indirect interdependencies among interacted systems/units [87]. For a set of time series {*s*_*i*_(*n*)}, *i* = 1,2,…, *L*, a matrix is defined with PSIs of bivariate signals as

(39) $$R=\left(\begin{array}{cccc} 1 & \lambda_{s_{1}s_{2}} & \ldots & \lambda_{s_{1}s_{L}}\\ \lambda_{s_{1}s_{2}}^{*} & 1 & \ldots & \lambda_{s_{2}s_{L}}\\ \vdots & \vdots & \ddots & \vdots \\ \lambda_{s_{1}s_{L}}^{*} & \lambda_{s_{2}s_{L}}^{*} & \ldots & 1 \end{array}\right).$$

Let Γ = **R** ^−1^ denote the inverse matrix of **R**. Then a measure called partial PSI is defined as

(40) $$\lambda_{ij\;|\;Z}=\frac{\left|\Gamma_{ij}\right|}{{\left[\Gamma_{ii}\Gamma_{jj}\right]}^{1/2}},$$

for {*s*_*i*_(*n*)} and {*s*_*j*_(*n*)}, conditioning on the remaining processes {*S*_*Z*_ | *Z* = 1,2,…, *L*, *Z* ≠ *i*, *j*} [87]. A partial PSI *λ*_*ij*∣*Z*_ ≈ 0 would imply that the association is induced by indirect coupling if the pair-wise PSI *λ*_*s*_*i*_*s*_*j*__ between {*s*_*i*_(*n*)} and {*s*_*j*_(*n*)} is significant.

## 6. Discussions and Conclusions

We give a technical review on PS analysis in this paper. In particular, we discuss IP definitions, PSI estimation and its significance test, the issues that may affect PS detection, and extensions of PSI. PS analysis is a method to quantify the mutual rhythmic interaction of coupled systems/units and has been used to infer functional connectivity from observed neural signals such as EEG and MEG. The main advantage of PS analysis is that it could detect weak interaction between signal pairs by only taking the IPs of signals into consideration but neglecting the influence of instantaneous amplitudes of signals. In addition, PS analysis could work for nonstationary signals. These merits imply that PS analysis is suitable for neuroscience research, as we are usually interested in the relationship between neural oscillations in particular frequency bands such as beta waves ([12, 30] Hz) and gamma waves ([30, 80] Hz) rather than the interaction between broadband raw signals.

While inferring functional neural connectivity with PS analysis, several cautions and limitations should be taken into consideration. First, the observed neural signal is usually with broadband spectra and unavoidably contaminated by noise. In this case, bandpass filter should be used to extract neural oscillations in the raw signals. Second, for spatially adjacent neural recordings, PS would be affected by volume conduction and so did correlation coefficient and mutual information. In this case, the phase lag index [76] or the weighted phase lag index [70] is recommended. Third, PS analysis does not work in a black-box way. The PSIs estimated with different durations of neural signals are not recommended to be compared. Fourth, users should be aware that PSI quantifies the variation instead of the mean of IP difference within a period. Therefore, PS analysis might not be suitable for analyzing those relatively stable components in neural signals, such as event-related potentials estimated from multiple EEG trials, as PS would ignore the amplitudes of components in event-related potential and miss the latencies of these components as well.

Various measures, such as cross-correlation, coherence, nonlinear interdependence [10], mutual information [8], partial directed coherence [9], correlation-entropy coefficient [92], and coherence entropy coefficient [13], have been proposed in functional connectivity analysis based on EEG, MEG signals and simulated data from various aspects [10–13, 93–95]. Results show that they can reveal a similar tendency of global connectivity [10]. The MPC-based PSI has been argued to be slightly better than coherence for both estimation and detection purposes [93]. In another study, total 34 different measures are classified into several families such as correlation/coherence family, mutual information family, PS family, and the Granger causality family. These measures are comprehensively compared, and results show that PS measures, Granger's causality measures and stochastic event synchrony measures are only weakly correlated with correlation coefficient, and are mutually uncorrelated as well [13]. These results imply that these measures each could characterize interdependence of signals from different aspects. With this consideration, we could have a good characterization of functional connectivity between neural signals with only a few representative measures of different families.
